# Enhancing radiative heat transfer with meta-atomic displacement

**DOI:** 10.1515/nanoph-2024-0729

**Published:** 2025-03-20

**Authors:** Cheng-Long Zhou, Shuihua Yang, Yang Huang, Yong Zhang, Hong-Liang Yi, Mauro Antezza, Cheng-Wei Qiu

**Affiliations:** School of Energy Science and Engineering, Harbin Institute of Technology, Harbin 150001, China; Key Laboratory of Aerospace Thermophysics, Ministry of Industry and Information Technology, Harbin, China; Department of Electrical and Computer Engineering, National University of Singapore, Singapore 117583, Singapore; School of Science, Jiangnan University, Wuxi 214122, China; Institut Universitaire de France, 1 rue Descartes, F-75231 Paris, France; Laboratoire Charles Coulomb (L2C), UMR 5221 CNRS-Universit de Montpellier, F-34095 Montpellier, France

**Keywords:** thermophotonics, heat transfer, meta-atomic displacement

## Abstract

Controlling and manipulating radiative heat transfer remains a pivotal challenge in both scientific inquiry and technological advancement, traditionally tackled through the precise geometric design of metastructures. However, geometrical optimization cannot break the inherent shackles of local modes within individual meta-atoms, which hinders sustained progress in radiative heat transfer. Here, we propose a comprehensive strategy based on interatomic displacement to achieve superior heat transfer performance while obviating the need for increasingly complex structural designs. This meta-atomic displacement strategy enables a shift from quasi-isolated localized resonances to extended nonlocal resonant modes induced by strong interactions among neighboring meta-atoms, resulting in a radiative heat conductance that surpasses other previously reported geometrical structures. Furthermore, this meta-atomic displacement strategy can be seamlessly applied to various metastructures, offering significant implications for advancing thermal science and next-generation energy devices.

## 1 Introduction

**Figure 1:**
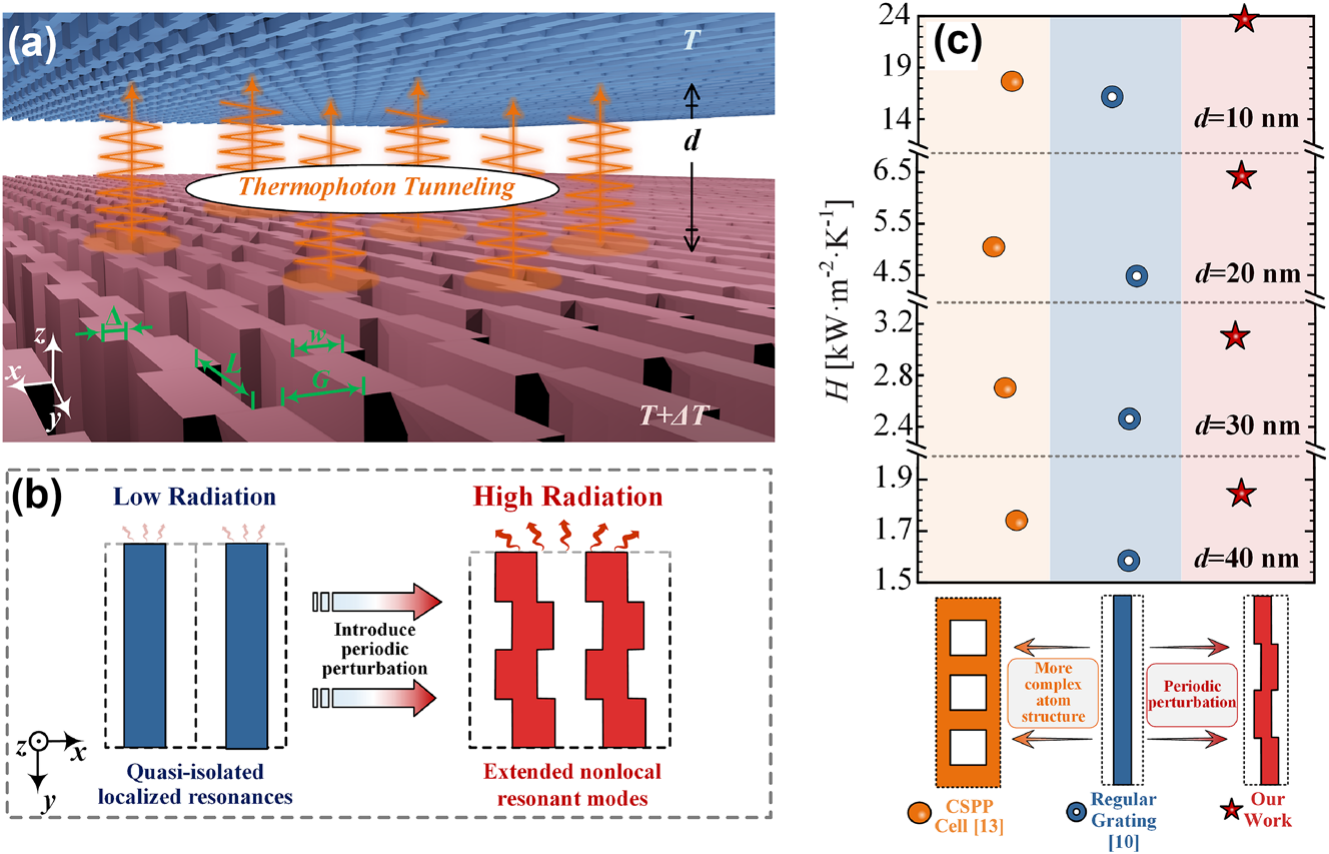
Structural and energetic properties of interatom-displacement-driven thermophotonic metasurface. (a) Schematics of RHT between two metastructures separated by a vacuum gap *d*, which have temperatures *T* and *T* + Δ*T*, respectively. (b) Conceptual diagram to design thermophotonic metastructures, illustrating the differences between the traditional and proposed approaches. The traditional approaches focus on the independent electromagnetic behavior of meta-atoms. In contrast, the proposed approaches rely on periodic displacement between meta-atoms to introduce additional interaction effects, stimulating stronger collective electromagnetic modes. (c) The heat transfer coefficient *H* for the regular grating, the more complex meta-atoms (cavity surface-plasmon polaritons [CSPPs] [13]), and proposed metastructures with strengthened interatom displacement, at different gaps.

Radiative heat transfer (RHT) is ubiquitous in nature, spanning from gigantic galaxies to microscopic atomic structures [1], [2], [3]. Effective manipulation of RHT is vital for mitigating diverse challenges such as global climate change [4], [5] and the overheating of electronics [6]. In this context, the question regarding the fundamental limits of RHT is attracting a lot of attentions. Since then, researchers investigated RHT in a variety of systems with objects of different shapes and materials, in pursuit of optimal radiative strategies [7], [8]. Among them, thermophotonic metastructurals are considered the most promising strategy [9]. Conventionally, the thermophotonic metastructures focus on the structural design of individual meta-atoms, trying to continuously optimize the local response of single meta-atom to pursue higher radiation performance [10], [11], [12], as schematically shown in Figure 1. For instance, Fernández-Hurtado et al. achieved much greater room-temperature radiative heat conductance than any unstructured material to date by constructing Si-based metastructures featuring two-dimensional periodic arrays of holes [13]. Motivated by the extraordinary effects, increasingly intricate micro- and nanostructures have sprung up, expanding the family of thermophotonic metastructures [14], [15], [16].

Nevertheless, the complexity of these meta-atoms is both a blessing and a curse. As the most straightforward approach, enhancing radiative heat transfer by persistently refining more intricate metacellular architectures is undoubtedly feasible [17]. Unfortunately, constrained by the degrees of freedom of the local modes, the independent response of each meta-atom cannot achieve a sustainable improvement with the increase in structural complexity [18], [19], [20]. The potential for further enhancing radiative heat transfer performance would diminish as the complexity of the meta-atom further increases. With the advancement in electronics and energy technologies, there is an increasing need for heat transfer performance [21], [22]. However, the current study of thermophotonic metastructures is mostly limited to the investigation of the local response of a single meta-atom, which cannot support further development of radiative heat transfer. Therefore, overcoming the current bottleneck in developing thermophotonic metastructures and finding a new general strategy to improve radiative heat transfer remains a formidable challenge.

In response to this challenge, we resort to displacement between meta-atoms to achieve an extraordinary thermal response, marking the first demonstration of the effects of meta-atomic displacement in RHT, as schematically depicted in Figure 1b. Utilizing rigorous coupled wave analysis, it is demonstrated that introducing inter-element displacement into traditional metastructures could markedly amplify radiative energy transfer, while surpassing conventional approaches that rely solely on optimizing meta-atom configurations. This also shows that the inter-element displacement effects are not just carriers of thermal information (as previously reported [23], [24]), but can help better manipulate thermal energy transfer. We then develop a nonlocal effective medium approach to predict non-trivial fingerprint of this thermo-metastructures, and demonstrate rigorously that the underlying physical mechanism responsible for this remarkable behavior is the existence of nonlocal electromagnetic response mode enabled by meta-atomic displacement. Moreover, we further demonstrate this inter-element displacement effect allows us to achieve a much higher radiative thermal conductivity than other metastructure to date, almost a factor of two higher than the metastructures with the previously reported maximum.

## 2 Extraordinary energy feature in interatom displacement

To illustrate our general strategy, first we concentrate on an instance of two mirrored metastructures formed by 2D alternating arrays on a semi-infinite planar substrate (see Figure 1b). Two mirrored metastructures are separated by a vacuum gap *d*. A conventional subwavelength grating comprises alternating strips of the core with a width *w* and the cladding groove with a width *G*, arrayed with a subwavelength period *P* = *G* + *w* along the direction perpendicular to the strips (*x*-axis). Referring to Figure 1b, in the proposed metastructures, the nanostrips are periodically partitioned into rectangular nanoblocks with a pitch *L* along the *y*-axis. The rectangular nanoblocks are then periodically dislocated by a distance Δ/2 in the *x*-direction. This dislocation introduces meta-atomic displacement of configuration assignment into the regular grating, which in turn enhances the interactions between the meta-atoms. The thickness of nonlocal metasurface is fixed at 200 nm. For the simplicity of analysis, the dielectric function of the substrate is set to 1. The filling ratio can be defined as *f* = *w*/*P*. Since the width of each strip and the dimension of the period along the *x* direction are the same, the structural displacement does not affect the filling ratio of the strips in the metastructures. The nanostrips are constructed from silicon (Si) with a doping concentration of 10^20^cm^−3^.

Theoretically, we combine fluctuational electrodynamics (FED) [25], [26] and rigorous coupled wave analysis (RCWA) [27], [28], [29] to reveal an radiative thermal effect of this metastructures. Our main goals focus on the analysis of the heat transfer coefficient (HTC), i.e. the radiative thermal conductance per unit area, at room temperature (300 K). In the framework of FED, the HTC between two arbitrary periodic metastructures can be expressed as follows [30]

(1) $$\begin{array}{cc} H & =\overset{\infty }{\underset{0}{\int }}h\left(\omega \right)d\omega \\ & =\overset{\infty }{\underset{0}{\int }}\frac{\partial \Theta \left(\omega ,T\right)}{\partial T}d\omega \overset{\pi /P}{\underset{-\pi /P}{\int }}\overset{\pi /P}{\underset{-\pi /P}{\int }}\frac{\xi dk_{x}dk_{y}}{{\left(2\pi \right)}^{3}}, \end{array}$$

where, *h*(*ω*) is the spectral heat transfer coefficient. $\Theta \left(\omega ,T\right)=\hbar \omega /\left(e^{\hbar \omega /k_{b}T}-1\right)$ is the average energy of a photon at frequency *ω* and temperature *T*, and *k*_*b*_ is the Boltzmann constant [31], [32]. *k*_*x*_, *k*_*y*_ and *k*_*z*_ denote the *x*, *y* and *z* components of the wavevector, with $k=\sqrt{k_{x}^{2}+k_{y}^{2}}$ representing its in-plane wavevector magnitude. The *ξ* represents the tunnelling probability of a thermal photon from the hot terminal to the cold terminal. Moreover, the thermophotons tunnelling probability can be given by RCWA (see Supplementary material (SM) [33] for explicit and rather standard expressions).

Let us start the discussion of the results by illustrating the main finding of our work. Figure 1c describes the room-temperature HTC versus the gap size for three metastructures with *P* = 50 nm and *f* = 0.4. This result is compared with the HTC for the regular grating and more complex meta-atoms [cavity surface-plasmon polaritons (CSPPs) structure] [13] with the same *P* and *f*. It is worth noting that when the Si plate is patterned as a regular grating, its HTC in the deep near-field regions (*d* < 50 nm) is already very significant, several times larger than the corresponding result for Si plates, as has been confirmed in many studies [10], [16]. In order to achieve a further breakthrough in radiative heat transfer, the structure of CSPPs meta-atom has been proposed [13]. As shown in Figure 1c, the HTC in both deep near-field regions is significantly improved when the meta-atoms structure is converted from the conventional grating to this CSPPs metacells.

Intriguingly, a more pronounced increase in radiative heat transfer is observed upon introducing a meta-atomic displacement, as seen in Figure 1c. This can be attributed to the system geometry, i.e., this interatom displacement introduces additional interactions into the system and optimizes the collective response behavior, thereby improving the radiative heat transfer performance. Taking *d* = 20 nm as an example, the HTC enhancement due to the meta-atomic displacement effect is 400 % of the increase amplitude from conventional idea of designing the metacell as CSPPs structure. However, it should be noted that there is a limit to this enhancement, and the enhancement resulting from this meta-atomic displacement disappears when the spacing is too large (see Section III of the SM [33] for details).

**Figure 2:**
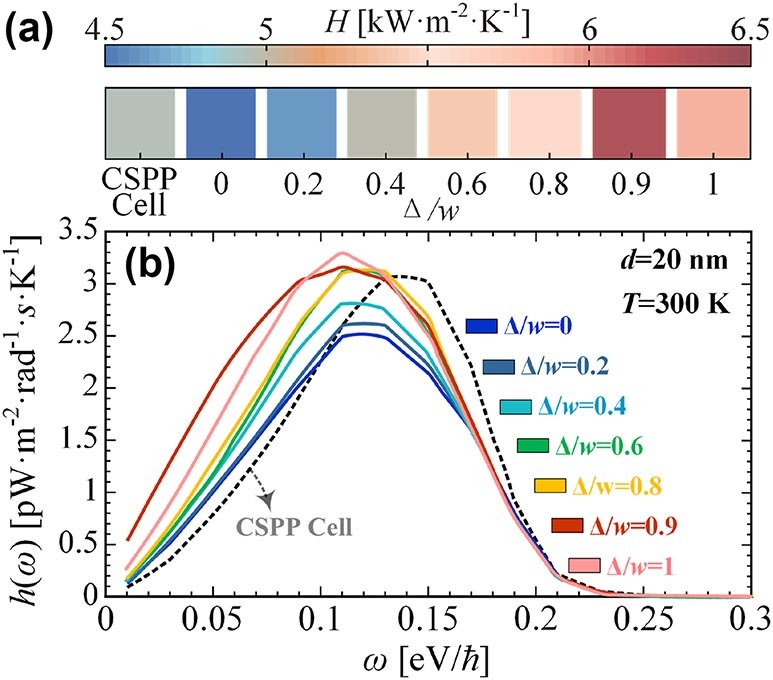
Heat transfer enhancement induced by the interatom displacement effect. (a) The heat transfer coefficient *H* versus the meta-atomic displacement degree Δ. (b) The spectral heat transfer coefficient *h*(*ω*) as a function of the frequency *ω*. The different lines correspond to different meta-atomic displacement degree Δ.

As shown in Figure 2a, the radiative heat transfer has a pronounced sensitivity to meta-atomic displacement. An increase in the radiative heat flux of the metastructures is observed when a small meta-atomic displacement (Δ/*w* = 0.2) is introduced. Upon reaching a degree of meta-atomic displacement of 0.9 Δ/*w*, the radiative heat transfer attains its maximum, exceeding that of a regular grating by 40 % and that of a CSPPs meta-atom by 28 %. Nevertheless, further reinforcement of the meta-atomic displacement cannot provide a sustained enhancement of the RHT. As the degree of misalignment increases above 0.9 Δ/*w*, or the two Si blocks are completely separated (*δ* = *w*), it can be observed that radiative heat transfer experiences a significant decline. This nonlinear enhancement indicates the complex wave response mechanisms in our thermo-metastructures, similar to optical [34], [35], acoustic [36], and other metastructures with strong interatom interactions.

To clarify the excellent properties of this interatom displacement metastructure, we present the spectral heat transfer coefficient in Figure 2b. It is noticed that the meta-atomic displacement does not affect the polarisation distribution of the system, which is still dominated by TM waves at this point, and a detailed analysis can be found in SM [33]. This spectral heat transfer coefficient indicates the energy levels carried by thermal photons of different frequencies. It can be seen that meta-atomic displacement broaden the spectral bandwidth while significantly intensifying the spectral peaks. As demonstrated in Figure 2b, increasing Δ/*w* from 0 to 0.9 results in a 28 % heightening in the spectral heat transfer coefficient (from 2.5 to 3.2 pW m^−2^ rad^−1^ s K^−1^). The meta-atomic displacements cannot result in a significant shift of the spectral peak. As Δ/*w* increases from 0 to 1, the frequency of spectral peak remains within the range of 0.1–0.13 eV/ℏ. The results reveal crucial significance that it demonstrates this interatom-dislocated metastructure can play crucial role in thermophotovoltaics [37] and electroluminescent refrigeration [6]. This feature enhances the power of the mentioned apparatus while maintaining optimal efficiency.

## 3 Local-nonlocal transition of thermophotons mode

**Figure 3:**
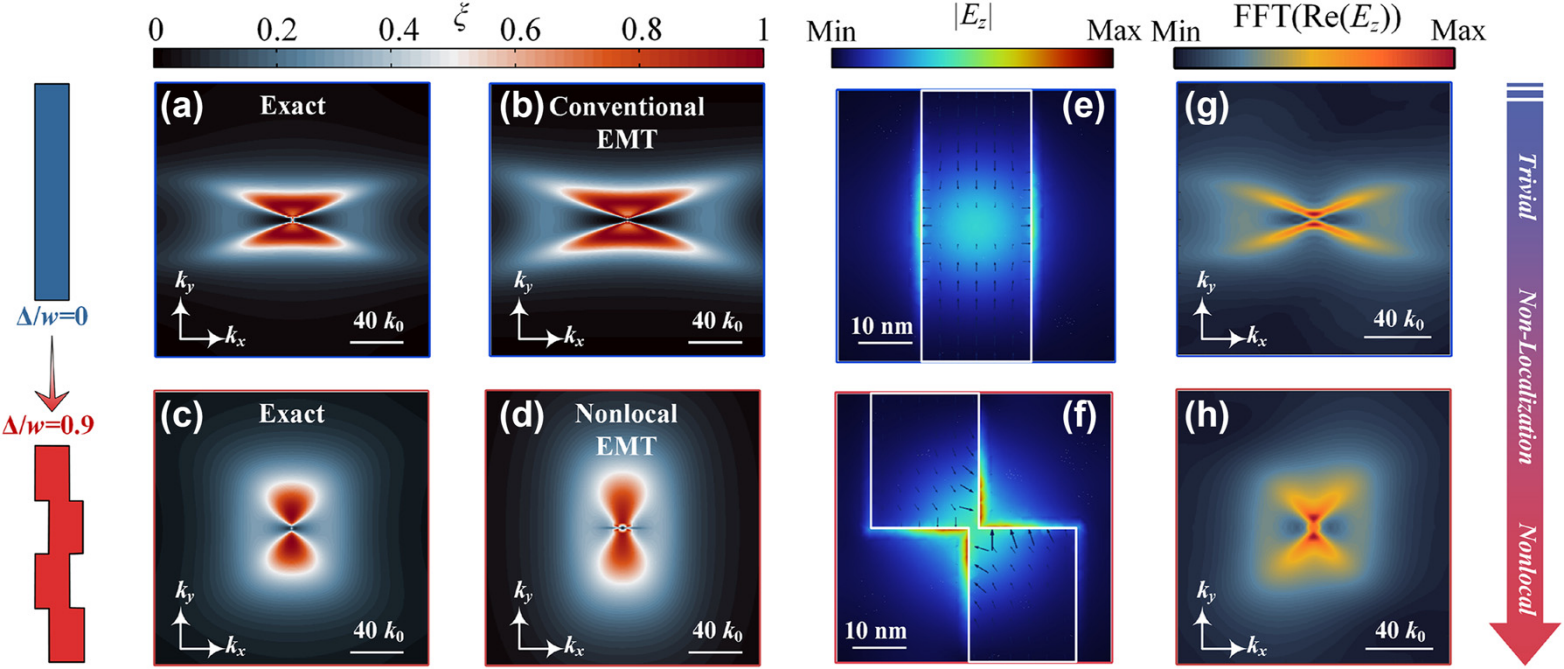
The thermophotonic tunnelling probability of regular grating (without meta-atomic displacement) for (a) exact solution (RCWA) and (b) EMT solution. The thermophotonic tunnelling probability of proposed thermo-metastructures (with meta-atomic displacement) for (c) exact solution (RCWA) and (d) EMT solution. The nonlocal effective model is shown in Eq. (2) (see also Ref. SM for the parameters). Electric field profiles (|*E*_*z*_|) of meta-atom with displacements of (e) Δ/*w* = 0 and (f) Δ/*w* = 0.9. The frequency is fixed at 0.11 eV/ℏ. The evolution of electric field distributions|*FFT*(*E*_*z*_)| of metastructure in response to different meta-atomic displacements in momentum space: (g) Δ/*w* = 0 and (h) Δ/*w* = 0.9. The field is excited by a dipole polarized along *z* placed 20 nm above the metastructure.

The thermophotons tunnelling probability indicates the tunneling probability of thermal photons between the emitter and receiver. *k*_0_ = *ω*/*c* being the wavenumber in vacuum. We also depict the thermophotons tunnelling properties of the regular grating for comparison in Figure 3a. It can be observed that the thermophotons tunnelling of the system exhibits a clear hyperbolic character, which is also consistent with previous studies [38], [39], [40]. In addition to the exact RCWA theory, we also employ the local effective medium theory (EMT) to facilitate the analysis. This approach treats the nanostructures as equivalent homogeneous biaxial plates, offering a computationally simple and rapid solution [14]. The effective dielectric function [*ɛ*_*xx*,emt_, *ɛ*_*yy*,emt_, *ɛ*_*zz*,emt_] of regular grating can be expressed in SM [33]. The local EMT theory can accurately predict the thermophotons tunnelling properties, thereby indicating the localized nature of the mode of this metasurface.

It is noteworthy that a notable alteration of the thermophotons properties of the metasurface can be obtained by adding meta-atomic displacement (from hyperbolic to dumbbell-like) (see Figure 3c). It demonstrates that a larger Δ suppresses the wavevector region of stronger thermophoton tunnelling (*ξ* > 0.8). However, a more significant meta-atomic displacement expands the bright band of weak thermophoton tunnelling (*ξ* < 0.5) into a wider wavevector region, which effectively counteracts the recession of strong thermophoton tunnelling while intensifies the spectral heat flux of the metastructure. Moreover, conventional local EMT theory fails to predict the nonlocal behavior of this metastructure. To address this limitation, we propose a nonlocal EMT model to precisely characterize the thermophotons behavior in metastructures. Given the absence of straightforward analytical expressions for nonlocal corrections associated with meta-atomic displacement, we incorporate these nonlocal corrections into the EMT model using a Taylor series expansion as a reference. This approach significantly reduces the prediction error of the electromagnetic response, particularly in the context of introducing meta-atomic displacement to metamaterials [41].

(2) $$\frac{Re\left(\varepsilon_{ii}^{\mathrm{non}}\left(k_{ii}\right)\right)}{Re\left(\varepsilon_{ii}\right)}=\frac{a_{0}\left(1+a_{1}|k_{ii}/k_{0}{|}^{2}+a_{2}|k_{ii}/k_{0}{|}^{4}\right)}{1+b_{1}|k_{ii}/k_{0}{|}^{2}+b_{2}|k_{ii}/k_{0}{|}^{4}},$$

where, the subscript *i* represents the direction *x* and *y*. The mentioned nonlocal corrections can be applied to the imaginary and real parts of the equivalent permittivity along different directions (see SM [33] for parameter details). It can be observed that incorporating nonlocal corrections significantly improves the agreement between the EMT-predicted thermophoton tunnelling coefficients and the exact solution, as seen in Figure 3d. The strong nonlocal behavior of metastructures can be attributed to the phenomenon of continuous reconstruction of the electric field distribution, thereby facilitating a transition from quasi-isolated localized resonances to extended nonlocal modes (see Figure 3e and f). This extended nonlocal resonant modes between the meta-atoms induced by strong interunit interactions further contribute to a significant change in the behaviour of surface polariton (see Figure 3g and h).

## 4 Interatom displacement in thermophotonic metastructures

**Figure 4:**
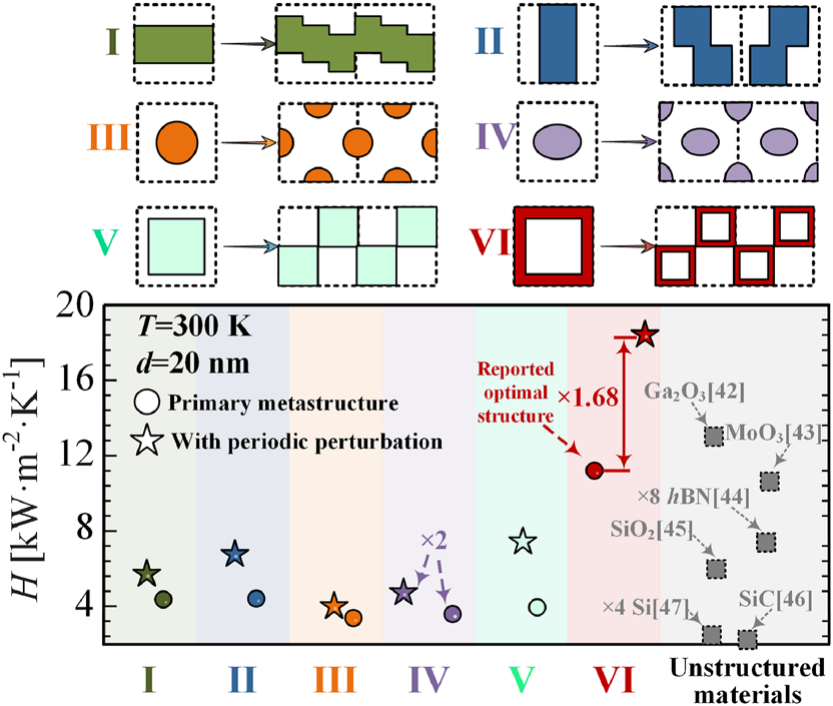
Dependence of the HTC on the introduction of meta-atomic displacement effect between meta-atoms for various designs (Si-based), which are labelled (I) to (VI). All square units have a 50 nm period. Furthermore, filling ratios remain consistent before and after the interactions effects. These designs and materials are regarded as promising candidates with the potential to exhibit high heat transfer performance [10], [13], [14], [40], [42], [43], [44], [45], [46], [47].

The presented strategy for enhancing radiative heat transfer by using periodic displacement between meta-atoms is not confined to a certain metasurface with the mentioned units. Instead, it is a general approach that can be employed for various thermophotonic metastructures, such as rectangular nanowires [10], circular nanorods [14], square nanorods [40], elliptical nanorods, square cavity [13], and many others, as shown in Figure 4 (the structural parameters can be seen as SM [33]). Silicon serves as the matrix material for these structures. These meta-atoms were previously regarded as artificial structures with exceptional radiative heat transfer performance. The square cavity structure, in particular, was previously considered to be a metastructures with room-temperature radiative thermal conductivity that can be much greater than any unstructured material [13]. Figure 4 indicates that when the distribution of meta-atoms is rearranged (i.e., a meta-atomic displacement is applied to the conventional distribution between the meta-atoms) to increase the interactions correlation between different elements, it leads to an overall increase in radiative heat transfer. Note that the reorganisation process preserves the original filling ratio *f* and the *f* is the optimum for the different structures. The HTC of cavity structure in Figure 4 is higher than that in Figure 1 due to the difference in *f* between the two. Surprisingly, the heat transfer coefficient at room temperature can reach up to 1.7 times that of previously reported structures at the highest level after interatom interactions enhancement of the square cavity structure. The strategy is equally effective for radiative enhancement at different temperatures, as can be seen in the SM [33].

Remarkably, our findings reveal that the introduction of meta-atomic displacement effects enable silicontraditionally regarded as a material with modest radiative propertiesto surpass high-performance materials such as Ga_2_O_3_ in radiative heat transfer. Notably, this photonics strategy is not limited to silicon but can also be extended to materials like Ga_2_O_3_, MoO_3_, and others, offering a versatile approach to significantly enhance their radiative heat transfer capabilities. Moreover, the primary focus of this work is on the formulation and validation of the concept of enhanced radiative heat transfer with displacement. Consequently, the global optimisation of the arrangement of structural unit is not involved in the aforementioned calculations. However, it is anticipated that the optimisation of the displacement of the structural units by certain global optimisation methodology will lead to further enhancement of the heat transfer performance [17], [48], [49]. Although it is possible to enhance the radiative heat transfer in the thermal metasurface with this method, there is still a considerable gap between the current HTC and the ideal HTC limit [50], [51], [52]. Let’s take the optimal bulk plasmonic material in Ref. [52] as an example, which at a vacuum gap of 20 nm is still close to three times that of the highest HTC in Figure 4.

## 5 Conclusions

We have proposed a conceptual framework to achieve unprecedented radiative heat transfer by exploiting interatomic displacement effects. This approach facilitates interactions among meta-atoms by introducing meta-atomic displacement that reconfigure structural periodicity, thereby enabling a transition from quasi-isolated localized resonances to extended nonlocal modes. Remarkably, the results reveal that this displacement-driven strategy can significantly amplify radiative heat transfer, yielding radiative heat conductances that surpass those of other proposed structures. These observed thermal responses suggest that radiative heat transfer can be effectively manipulated through introducing meta-atomic displacement effects into the distribution of meta-atoms, eliminating the need for increasingly complex metastructure designs. We contend that this approach offers a definitive pathway for advancing research in radiative heat transfer, paving the way for innovative applications in thermal management [53], thermophotovoltaics [37], photonic cooling [54], [55], thermocomputation [56], and near-field imaging [57].

## Supplementary Material

Supplementary Material Details
