# Supplementary material for: Enhancing radiative heat transfer with meta-atomic displacement
Source: Nanophotonics. 2025 Mar 20;14(23):4045–52. doi: 10.1515/nanoph-2024-0729 (PMC12617729; doi:10.1515/nanoph-2024-0729)
Supplement: Supplementary file 1 — Supplementary Material Details [file j_nanoph-2024-0729_suppl_001.docx]

**Supplemental Material:**

**Enhancing Radiative Heat Transfer with Meta-atomic Displacement**

Cheng-Long Zhou^1,2^, Shui-Hua Yang^3^, Huang Yang^4^, Yong Zhang^1,2^,

Hong-Liang Yi^1,2,†^, Mauro Antezza^5,6^, and Cheng-Wei Qiu^3, §^

^1^*School of Energy Science and Engineering*, *Harbin Institute of Technology*, *Harbin 150001*, *China*

^2^*Key Laboratory of Aerospace Thermophysics, Ministry of Industry and Information Technology, Harbin, China*

^3^*Department of Electrical and Computer Engineering, National University of Singapore, Kent Ridge 117583, Singapore*

^4^*School of sciencejiangnan university, Wuxi 214122, China*

*^5^School of Nano Science, Institute for Research in Fundamental Sciences (IPM),* *Tehran 19395–5531, Iran*

*^6^Institut Universitaire de France, 1 rue Descartes, F-75231 Paris, France*

S1. The computational details for the radiative heat transfer and the nonlocal effective medium theory

S2. Impact of temperature on the meta-atomic displacement effect

S3. The radiative heat transfer of designing thermophotonic metastructures at different structure parameters

S4. Thermal photons evolution in designing thermophotonic metastructures with different structure parameters

S5. The derivation and result of electromagnetic property with different structure parameters

Fig. S1 to S8

**S1. The computational details** **for the radiative heat transfer and the nonlocal effective medium theory**

In this section we show that our computational details for the radiative heat transfer and the nonlocal efficient medium theory. We solve Maxwell's equations for the monochromatic components of the electric fields $E_{\omega}\left(z\right)e^{ik_{\rho}r-i\omega t}$ and magnetic fields $B_{\omega}\left(z\right)e^{ik_{\rho}r-i\omega t}$ in a planar geometry with two metastructures separated by a vacuum gap *d* [S1]

$\left(ik_{\rho}+e_{z}\frac{\partial}{\partial z}\right)\times E_{\omega}=\frac{i\omega}{c}B_{\omega}$ (S1)

$\left(ik_{\rho}+e_{z}\frac{\partial}{\partial z}\right)\times B_{\omega}=\frac{4\pi}{c}\sum_{\alpha=E,R} \delta\left(z-z_{\alpha}\right)\left(j_{k_{\rho}\omega}^{\left(\alpha\right)}+\tilde{j}_{k_{\rho}\omega}^{\left(\alpha\right)}\right)-\frac{i\omega}{c}\varepsilon\left(z\right)E_{\omega}$ (S2)

where **e**_z_ is the unit vector in the *z* direction, perpendicular to the layers. The surface current in each layer *α* = 1,2 consists of two contributions: $j_{k_{\rho}\omega}^{\left(\alpha\right)}=\chi\left(\omega,T\right)E_{\omega}\left(z_{\alpha}\right)$ is the induced current due to the electric field, while the fluctuating currents $j_{k_{\rho}\omega}^{\left(\alpha\right)}=\left(j_{k_{\rho}\omega}^{\left(\alpha\right)}\right)^{*}$ are complex Gaussian random variables with the correlator determined by the fluctuation-dissipation theorem with the correlator determined by the fluctuation-dissipation theorem [S2]. The thermal radiation is generated by fluctuating current sources, where the magnitude of the current

$\left\langle j_{k_{\rho}\omega}^{\left(\alpha\right)}j_{-k_{\rho}-\omega}^{\left(\alpha'\right)}\right\rangle=\frac{4\varepsilon_{0}\omega}{\pi}\frac{\varepsilon_{\alpha\beta}-\varepsilon_{\beta\alpha}^{*}}{2i}\Theta\left(\omega,T\right)\delta\left(\mathbf{r}-\mathbf{r}'\right)\delta\left(z-z'\right)$ (S3)

where, $\mathbf{r}$and $\mathbf{r}'$ are the spatial vector, *α* and *β* are components in a Cartesian coordinate system, *ε* is element of the dielectric function, Θ(*ω*, *T*) =*ћ*ω/(*exp*(*ћω*/*k_b_T*)−1) is the average energy of a photon at frequency *ω* (the Boltzmann factor), and *δ*(*) is the Dirac function. Based on this framework, the solution of Maxwell's system of equations decouples into a transverse magnetic mode and a transverse electric mode, with their contributions to the thermal current being straightforwardly additive. In all the calculations that have been performed, the spatial coordinates in the plane have been limited to a single cell. To calculate the radiated power, it is first necessary to determine the ensemble-averaged Poynting vector along the *z*-direction. The integration method can be found in Ref. [S2]. The energy transport from, say, emitter to receiver is given by the average Joule loss power per unit area $H\left(T\right)=\left\langle\tilde{j}^{E}\cdot E^{E}\right\rangle_{T+\Delta T}-\left\langle\tilde{j}^{R}\cdot E^{R}\right\rangle_{T}$. For a temperature-independent relaxation time, this energy current can be shown as [S3]

$H=\int_{0}^{\infty} h\left(\omega\right)d\omega=\int_{0}^{\infty} \frac{d\omega}{2\pi}\frac{\partial\Theta\left(\omega,T\right)}{\partial T}\int_{0}^{\infty} \int_{0}^{\infty} \frac{dk_{x}dk_{y}}{\left(2\pi\right)^{2}}\xi\left(\omega,k_{x},k_{y}\right)$, (S4)

here, *k_x_* and *k_y_* are the surface wavevectors along the *x* and *y* axes, respectively. When the surface parallel wavevector $k=\text{(}k_{x}^{2}+k_{y}^{2}\text{)}^{1/2}$ is greater than the wavevector in vacuum, *k*_0_ = *ω*/*c*_0_, the electromagnetic wave excited by thermal energy takes the form of an evanescent wave. The *ξ* represents the tunnelling probability of a thermophoton from the hot terminal to the cold terminal. Within the RCWA approach, we express the fields in our periodic system as a sum of plane waves using the Bloch theorem. Thus, the tunnelling function above can be obtained by combining scattering matrices of the different interfaces in reciprocal space. If we place the coordinate origin at metastructure with higher temperature (label as *hot*), the thermophoton tunnelling probability can be expressed as

$\xi\left(\omega,k_{x},k_{y}\right)=\text{Tr}\left(\hat{D}\hat{W}_{hot}\hat{D}^{\dagger}\hat{W}_{cold}\right),$ (S5)

where

$\hat{D}=\left(\hat{1}-\hat{S}_{hot}\hat{S}_{cold}\right)^{-1},$ (S6)

$\hat{W}_{hot}=\hat{\sum}_{-1}^{\text{pw}}-\hat{S}_{hot}\hat{\sum}_{-1}^{\text{pw}}\hat{S}_{hot}^{\dagger}+\hat{S}_{hot}\hat{\sum}_{-1}^{\text{ew}}-\hat{\sum}_{-1}^{\text{ew}}\hat{S}_{hot}^{\dagger},$ (S7)

$\hat{W}_{cold}=\hat{\sum}_{+1}^{\text{pw}}-\hat{S}_{cold}\hat{\sum}_{+1}^{\text{pw}}\hat{S}_{cold}^{\dagger}+\hat{S}_{cold}\hat{\sum}_{+1}^{\text{ew}}-\hat{\sum}_{+1}^{\text{ew}}\hat{S}_{cold}^{\dagger},$ (S8)

here, $\hat{S}_{hot}=\hat{R}_{hot}$and $\hat{S}_{cold}=e^{ik_{z}d}\hat{R}_{cold}e^{ik_{z}d}$, in which $\hat{R}_{hot}$and $\hat{R}_{cold}$are the reflection matrices of the two vacuum-metastructure interfaces. $k_{z}=\text{(}k_{0}^{2}-k^{2}\text{)}^{1/2}$ is the wavevector component perpendicular to the metastructure interfaces. These matrices were computed with the scattering-matrix approach of Ref. [S2]. Moreover, the matrix is a projector into the propagating (evanescent) sector. All these matrices are 2*N* × 2*N* matrices, where *N* is the number of reciprocal lattice vectors included in the plane-wave expansions. The calculated transmission coefficient *ξ* would converge to a constant value with a sufficiently large diffraction order [S3]. To save computational costs and ensure numerical accuracy, the maximum diffraction order is iterated by increasing the order by 100 steps each time until the relative error of this diffraction order with respect to three times its diffraction order at a given frequency is less than 3%. Eventually, the maximum diffraction order *N* = 100 is used based on this convergence analysis.


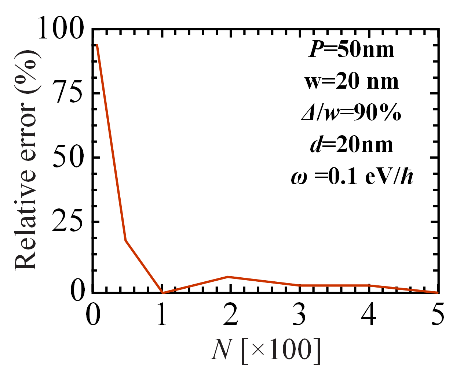


**Fig S1** Convergence test for thermophoton tunnelling probability with period *P*= 50 nm, vacuum gap *d* = 20 nm, and Δ/*w*=0.9.

On the other hand, the wavevector integral in Eq. (S4) must be calculated in the interval (-π/*a*, π/*a*) for both *k_x_* and *k_y_*. Moreover, in all our calculations, the in-plane spatial coordinates are restricted to the single unit cell. The dielectric function of doped Si is described within a Drude model [S4]: $\varepsilon_{Si}=\varepsilon_{\infty}-\omega_{p}^{2}/\left(\omega^{2}+i\gamma\omega\right)$, where$\varepsilon_{\infty}$=11.7, *ω_p_* = 715 meV is the plasma frequency, and *γ* = 60.2 meV is the damping. These values correspond to a doping level of 10^20^ cm^−3^.To better analyze the results, we have made used of an effective medium theory (EMT). Within this theory, our metastructure can be modeled as uniaxial materials with a diagonal permittivity tensor: $\hat{\varepsilon}=\left[\varepsilon_{xx,emt},\varepsilon_{yy,emt},\varepsilon_{zz,emt}\right]$, where the subindex *xx* (*yy*) and *zz* denote the ordinary and extraordinary optical axis, respectively. The components of the dielectric tensor are given by [S5]:

$\varepsilon_{xx,yy}=\varepsilon_{Si}\frac{\varepsilon_{Si}\left(1-f\right)+1+f}{\varepsilon_{Si}\left(1+f\right)+1-f},\varepsilon_{zz}=\varepsilon_{Si}\left(1-f\right)+f$ (S9)

Moreover, conventional EMT theory is unable to predict the thermophoton tunnelling behavior of nonlocal metastructure. In the context of introducing meta-atomic displacement for metamaterials, the prediction error in electromagnetic response can be substantially diminished by implementing suitable nonlocal corrections within the EMT model. This strategy can also be applied to thermophoton tunnelling of the meta-atomic displacement scenarios of interest here. However, there is no simple analytical expression for the nonlocal corrections about meta-atomic displacement. We use a numerical approach, the details of which have been shown in Eq. (2) the main text. Detailed details of the parameters of nonlocal correction are given in Table S1. In addition, since there is no meta-atomic displacement in the *z*-direction, there is no need for nonlocal corrections.

**Table S1** EMT non-local correction parameters

| *xx* | *a*_0_ | *a*_1_ | *a*_2_ | *b*_1_ | *b*_2_ |
| --- | --- | --- | --- | --- | --- |
| Real part | 2.25 | 0.15 | 1.725 | 2 | 0.95 |
| Imaginary part | 60 | 4 | 16 | 2 | 0.95 |
| *yy* | *a*_0_ | *a*_1_ | *a*_2_ | *b*_1_ | *b*_2_ |
| Real part | 0.3 | 8 | 0.16 | 0.95 | 1.1 |
| Imaginary part | 5.25 | 140 | 2.8 | 0.95 | 1.1 |

For the anisotropic plate structure, the reflection coefficient matrix **R** at the interface between air and anisotropic slab take the forms [S6]:

$R(k_{x},k_{y})=\left[\begin{matrix} r^{ss}(k_{x},k_{y}) & r^{sp}(k_{x},k_{y}) \\ r^{ps}(k_{x},k_{y}) & r^{pp}(k_{x},k_{y}) \end{matrix}\right]$ (S10)

The permittivity tensor in the *x*-*y*-*z* coordinate system can be expressed as

$\bar{\bar{\varepsilon}}(k_{x},k_{y})\text{=}\left[\begin{matrix} \varepsilon_{xx}(k_{x},k_{y}) & \varepsilon_{xy}(k_{x},k_{y}) & \varepsilon_{xz}(k_{x},k_{y}) \\ \varepsilon_{yx}(k_{x},k_{y}) & \varepsilon_{yy}(k_{x},k_{y}) & \varepsilon_{yz}(k_{x},k_{y}) \\ \varepsilon_{zx}(k_{x},k_{y}) & \varepsilon_{zy}(k_{x},k_{y}) & \varepsilon_{zz}(k_{x},k_{y}) \end{matrix}\right].$ (S11)

For the TM wave, the electromagnetic fields in the anisotropic medium can be expressed with reference to the *x*-*y*-*z* coordinate system as the following form:

$H=U(z)exp\left(i\omega\tau-ikx\right)$ (S12)

$E=i\sqrt{\frac{\mu_{0}}{\varepsilon_{0}}}S(z)exp\left(i\omega\tau-ikx\right)$ (S13)

here, *k* is the surface parallel wavevector, *ε*_0_ is the vacuum permittivity, and *μ*_0_ is the vacuum permeability. Substituting Eqs. (S11)-(S13) into the Maxwell equations and setting *K=k/k*_0_, the differential equations can be expressed as [S6]

$\frac{d}{dz}\left(\begin{matrix} S_{x} \\ S_{y} \\ U_{x} \\ U_{y} \end{matrix}\right)=k_{0}A\left(\begin{matrix} S_{x} \\ S_{y} \\ U_{x} \\ U_{y} \end{matrix}\right)$ (S14)

where

$A=\left[\begin{matrix} iK\frac{\varepsilon_{zx}(k_{x},k_{y})}{\varepsilon_{zz}(k_{x},k_{y})} & iK\frac{\varepsilon_{zy}(k_{x},k_{y})}{\varepsilon_{zz}(k_{x},k_{y})} & 0 & \frac{K^{2}}{\varepsilon_{zz}(k_{x},k_{y})}-1 \\ 0 & 0 & 1 & 0 \\ \frac{\varepsilon_{yz}(k_{x},k_{y})\varepsilon_{zx}(k_{x},k_{y})}{\varepsilon_{zz}(k_{x},k_{y})}-\varepsilon_{yx} & \frac{\varepsilon_{yz}(k_{x},k_{y})\varepsilon_{zy}(k_{x},k_{y})}{\varepsilon_{zz}(k_{x},k_{y})}+K^{2}-\varepsilon_{yy}(k_{x},k_{y}) & 0 & -iK\frac{\varepsilon_{yz}(k_{x},k_{y})}{\varepsilon_{zz}(k_{x},k_{y})} \\ \varepsilon_{xx}-\frac{\varepsilon_{xz}\varepsilon_{zx}}{\varepsilon_{zz}} & \varepsilon_{xy}-\frac{\varepsilon_{xz}\varepsilon_{zy}}{\varepsilon_{zz}} & 0 & -iK\frac{\varepsilon_{xz}}{\varepsilon_{zz}} \end{matrix}\right]$ (S15)

The eigenvalues and eigenvectors of the coefficient matrix ***A*** can describe effectively the electromagnetic fields in the anisotropic medium. We define the thickness of this anisotropic medium as *t*. The electromagnetic field vector components in the anisotropic slab with a thickness *t* can be expressed as [S6]

$S_{x}\left(z\right)=\sum_{m=1}^{2} w_{1,m}c_{m,+}\exp\left(k_{0}q_{m}z\right)+\sum_{m=1}^{2} w_{1,m+2}c_{m,-}\exp\left(k_{0}q_{m}\left(z-t\right)\right)$ (S16)

$S_{y}\left(z\right)=\sum_{m=1}^{2} w_{2,m}c_{m,+}\exp\left(k_{0}q_{m}z\right)+\sum_{m=1}^{2} w_{2,m+2}c_{m,-}\exp\left(k_{0}q_{m}\left(z-t\right)\right)$ (S17)

$U_{x}\left(z\right)=\sum_{m=1}^{2} w_{3,m}c_{m,+}\exp\left(k_{0}q_{m}z\right)+\sum_{m=1}^{2} w_{3,m+2}c_{m,-}\exp\left(k_{0}q_{m}\left(z-t\right)\right)$ (S18)

$U_{y}\left(z\right)=\sum_{m=1}^{2} w_{4,m}c_{m,+}\exp\left(k_{0}q_{m}z\right)+\sum_{m=1}^{2} w_{4,m+2}c_{m,-}\exp\left(k_{0}q_{m}\left(z-t\right)\right)$ (S19)

here, *w_i,m_* and *q_m_* are the element of the eigenvector matrix ***W*** and the eigenvalue of matrix ***A***, respectively. Noticed that the real parts of *q*_1_ and *q*_2_ are negative. *c_m_*_,+_ and *c_m_*,- are the unknowns and can be determined by applying the boundary conditions. The reflection and transmission coefficients can be calculated by matching the tangential electric and magnetic field components at the top surface of the slab as

$\left(\begin{matrix} -\frac{ik_{z0}}{k_{0}} \\ 0 \\ 0 \\ 1 \end{matrix}\right)+\left(\begin{matrix} \begin{matrix} \frac{ik_{z0}}{k_{0}} \\ 0 \\ 0 \\ 1 \end{matrix} & \begin{matrix} 0 \\ -i \\ \frac{k_{z0}}{k_{0}} \\ 0 \end{matrix} \end{matrix}\right)\left(\begin{matrix} r^{pp}(k_{x},k_{y}) \\ r^{ps}(k_{x},k_{y}) \end{matrix}\right)=\left[\begin{matrix} W_{1} & W_{2}X \end{matrix}\right]\left[\begin{matrix} C_{+} \\ C_{-} \end{matrix}\right]$ (S20)

and at the bottom surface of the slab as

$\left[\begin{matrix} W_{1} & W_{2}Y \end{matrix}\right]\left[\begin{matrix} C_{+} \\ C_{-} \end{matrix}\right]=\left(\begin{matrix} \begin{matrix} -\frac{ik_{z0}}{k_{0}} \\ 0 \\ 0 \\ 1 \end{matrix} & \begin{matrix} 0 \\ -i \\ -\frac{k_{z0}}{k_{0}} \\ 0 \end{matrix} \end{matrix}\right)\left(\begin{matrix} t^{pp}(k_{x},k_{y}) \\ t^{ps}(k_{x},k_{y}) \end{matrix}\right)$ (S21)

here, ***W***=[***W***_1_ ***W***_2_] is the eigenvector matrix of matrix ***A****.* ***C***_+_ and ***C***- are the vectors composed of the unknowns, ***X*** is a diagonal matrix with the diagonal elements as exp(-*k*_0_*q_m_t*), *m*=3,4. ***Y*** is a diagonal matrix with the diagonal elements as exp(-*k*_0_*q_m_t*), *m*=1,2. $k_{z0}\text{=(}k_{0}^{2}-k^{2}\text{)}^{1/2}$ is the tangential wavevector along the *z* direction in vacuum. When the incident wave is a TE wave, the electromagnetic fields in the anisotropic medium can be written as follows

$E=S(z)exp\left(i\omega\tau-ikx\right)$ (S22)

$H=-i\sqrt{\frac{\varepsilon_{0}}{\mu_{0}}}U(z)exp\left(i\omega\tau-ikx\right)$ (S23)

The calculation of the TE wave is similar to that of the TM wave above. Substituting Eqs. (S22) and (S23) into the Maxwell equations can obtain the expression of the electromagnetic fields with the same forms as in Eqs. (S16)–(S19). The reflection and transmission coefficients can be calculated by matching the tangential electric and magnetic field components at the top surface of the slab as

$\left(\begin{matrix} 0 \\ 1 \\ -\frac{ik_{z0}}{k_{0}} \\ 0 \end{matrix}\right)+\left(\begin{matrix} \begin{matrix} -\frac{k_{z0}}{k_{0}} \\ 0 \\ 0 \\ i \end{matrix} & \begin{matrix} 0 \\ 1 \\ \frac{ik_{z0}}{k_{0}} \\ 0 \end{matrix} \end{matrix}\right)\left(\begin{matrix} r^{sp}(k_{x},k_{y}) \\ r^{ss}(k_{x},k_{y}) \end{matrix}\right)=\left[\begin{matrix} W_{1} & W_{2}X \end{matrix}\right]\left[\begin{matrix} C_{+} \\ C_{-} \end{matrix}\right]$ (S24)

and at the bottom surface of the slab as

$\left[\begin{matrix} W_{1} & W_{2}Y \end{matrix}\right]\left[\begin{matrix} C_{+} \\ C_{-} \end{matrix}\right]=\left(\begin{matrix} \begin{matrix} \frac{k_{z0}}{k_{0}} \\ 0 \\ 0 \\ \begin{matrix} \\ i \end{matrix} \end{matrix} & \begin{matrix} 0 \\ 1 \\ -\frac{ik_{z0}}{k_{0}} \\ 0 \end{matrix} \end{matrix}\right)\left(\begin{matrix} t^{sp}(k_{x},k_{y}) \\ t^{ss}(k_{x},k_{y}) \end{matrix}\right)$ (S25)

In addition, the structural parameters of the various thermophotonic metastructures (proposed in Fig. 4 of main text) are also given in this section. It is worth noting that the reorganization process does not change the original filling ratio. All configurations have a period of 50 nm. Detailed details of the parameters of these thermophotonic metastructures are given in Table S2.

**Table S2** Detailed details of the parameters of the various thermophotonic metastructures

| *Configuration I* | | *Configuration II* | | *Configuration III* | |
| --- | --- | --- | --- | --- | --- |
| ΔI | 14 nm | ΔII | 16 nm | *R* | 14.14 nm |
| *Configuration IV* | | *Configuration V* | | *Configuration VI* | |
| *a*/*b* | 14.14 nm/  7.07 nm | *w_V_* | 31.8 nm | *w_VI_* | 34.6 nm |


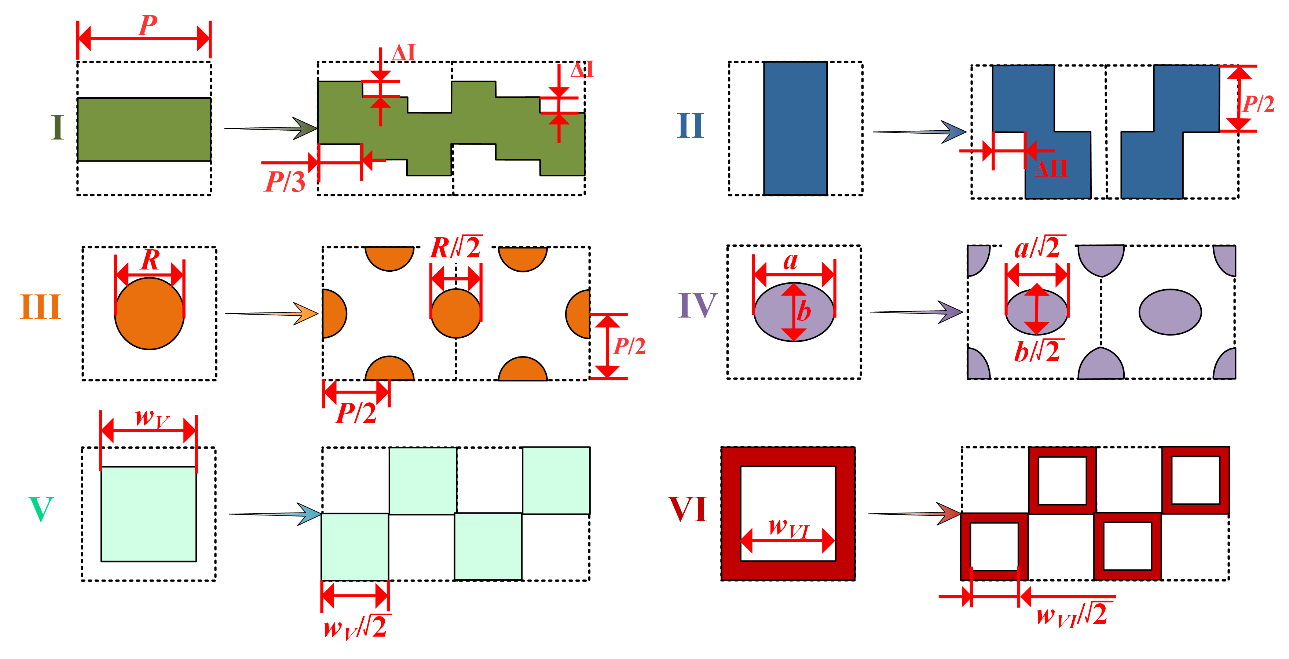


**Fig S2** Schematics of various thermophotonic metastructures proposed in Fig. 4 of main text.

**S2. Impact of temperature on the meta-atomic displacement effect**

In addition, the temperature can influence the RHF of the interactive thermo-metastructures by modifying the mean energy of a Planck oscillator $\Theta(\omega,T)$. In this section, we show the heat transfer coefficient at different temperatures for the structures that appear in the main text. The HTC at different temperatures is higher than that of the original grating structure both for the interactive structure proposed in the main text and for configuration I and configuration II structures. When the temperature reaches 1000 K, the increase in heat transfer compared to the original grating is still close to 40%. [see Fig. S3(a)]. Interestingly, Figure S3(b) show that for configuration III, the advantages of this interactive thermo-metastructure are even more pronounced in high temperature environments (the increase over its conventional configuration can be increased to 139% at 1000 K). For configuration IV, the benefits of this heat are maintained: the increase over its conventional configuration can be increased to 160% at 1000 K [see Fig. S3(c)]. For square-cavity (configuration V) and square-column metastructures, a significant heat transfer enhancement effect can still be obtained at high temperatures by disrupting their periodic arrangement [see Figs. S3(d) and S3(e)].


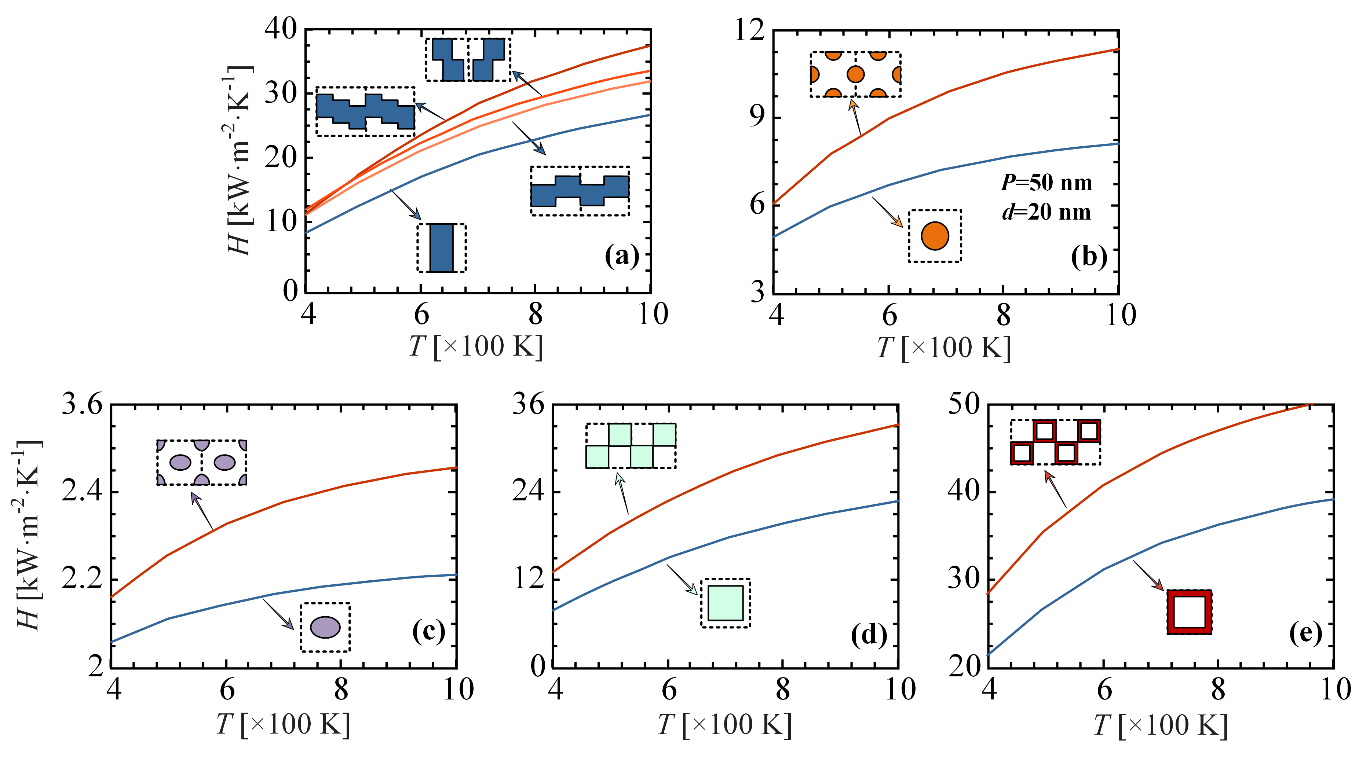


**Fig S3** Dependence of the HTC on the introduction of meta-atomic displacement effect between meta-atoms for various designs (proposed in main text) at different temperatures.

**S3. The radiative heat transfer of designing thermophotonic metastructures at different structure parameters**

In this section, the vacuum gap is fixed at 50 nm, and the period is fixed at 60 nm. Here, the width of alternating strips is first fixed at 20 nm. In the conventional local case (no meta-atomic displacements), the thermal conductivity can be close to 0.98 kW·m^-2^·K^-1^, which is about 4.1 times in magnitude larger than that of a silicon platen, as illustrated in Fig. S4(c). This also proves the excellent reinforcement effect of conventional metastructures for radiative heat transfer. Interestingly, it is notable that a pronounced increase in the radiative heat flux of the metastructures is observed when a meta-atomic displacement is introduced, as seen in Fig. S4(c). Upon reaching a degree of meta-atomic displacement of 0.9 Δ/*w*, it becomes evident that the radiative heat transfer of the metastructures attains its maximum, exceeding that of a conventional local metastructures by 20%. Nevertheless, the further reinforcement of the meta-atomic displacement could not result in a sustained enhancement of the radiative heat transfer of the system. As the degree of misalignment increases above 0.9 Δ/*w*, or the two silicon blocks are completely separated (Δ= *w*), it can be observed that radiative heat transfer experiences a significant decline.


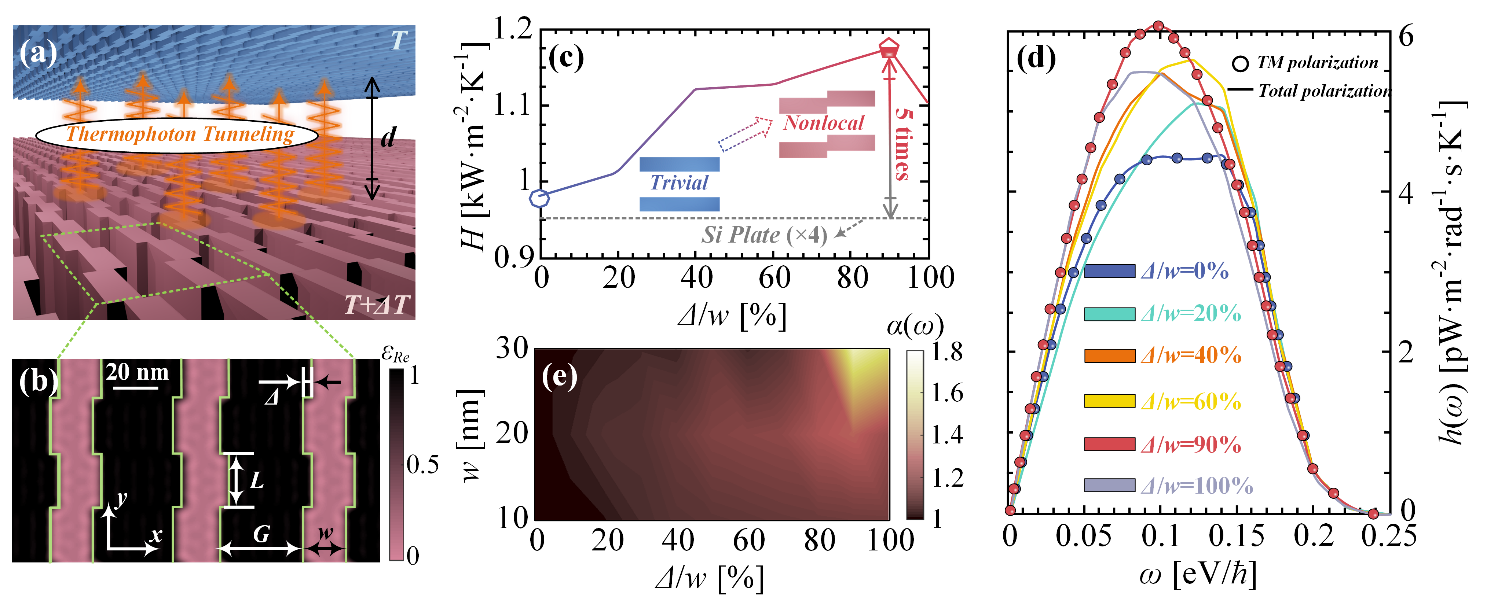


**Fig S4** (a) Schematics of radiative heat transfer between two metastructures separated by a vacuum gap *d*, both of which have temperatures *T* and *T*+*ΔT*, respectively. The vacuum gap is 50 nm, and the temperature is 300 K. (b) 2D Schematics of the metastructures. (c) The heat transfer coefficient *H* for various dislocated length of silicon segment Δ. The strip widths *w* is 20 nm. (d) The spectral heat transfer coefficient *h*(*ω*) as a function of the frequency *ω*. The different lines correspond to different dislocated lengths of silicon segment Δ. The line shows the contribution of total-polarized waves, and the circular mark shows the contribution of TM-polarized waves. (e) Enhancement rates *α*(*ω*) of spectral heat transfer coefficients for different dislocated lengths Δ at different strip widths *w*. The frequency is fixed at 0.1 eV/*ћ*.

To provide clarification of the excellent properties of heat transfer manipulation, we present the spectral heat transfer coefficient in Fig. S4(d). This spectral heat transfer coefficient indicates the energy levels carried by thermal photons of different frequencies. Meta-atomic displacements do not affect the spectral bandwidth, but significantly intensify the spectral peaks. As demonstrated in Fig. S4(d), increasing Δ/*w* from 0 to 0.9 results in a 39% heightening in the spectral heat transfer coefficient of the system (from 4.44 to 6.15 kW·m^-2^·K^-1^. The meta-atomic displacements do not result in a significant shift of the spectral peak. The frequency of spectral peak remains within the range of 0.08-0.13 eV/*ћ*, as Δ/*w* increases from 0 to 1. This feature has the potential to enhance the power of the aforementioned apparatus while maintaining optimal efficiency. To more clearly illustrate this spectral enhancement phenomenon, Fig. S4(e) depicts the enhancement rates *α* = *h*(Δ)/*h*(0) of spectral HTC for different dislocated lengths Δ with different strip widths *w*. The frequency is fixed at 0.1 eV/*ћ*. It can be observed that the spectral enhancement resulting from meta-atomic displacement can reach a maximum of 180% when the width is increased to 30 nm. Moreover, we also show in Fig. S4(d) the contribution of evanescent TM-polarized waves to the HTC of the doped-Si metastructures. Note that this contribution dominates the radiative heat transfer as Δ/*w* increases from 0 to 0.9. It also demonstrates that the radiative heat transfer of the system is predominantly excited by the surface modes rather than the magnetic polaritons, which are in a good agreement with the results obtained with Si-based metastructures featuring two-dimensional periodic arrays.


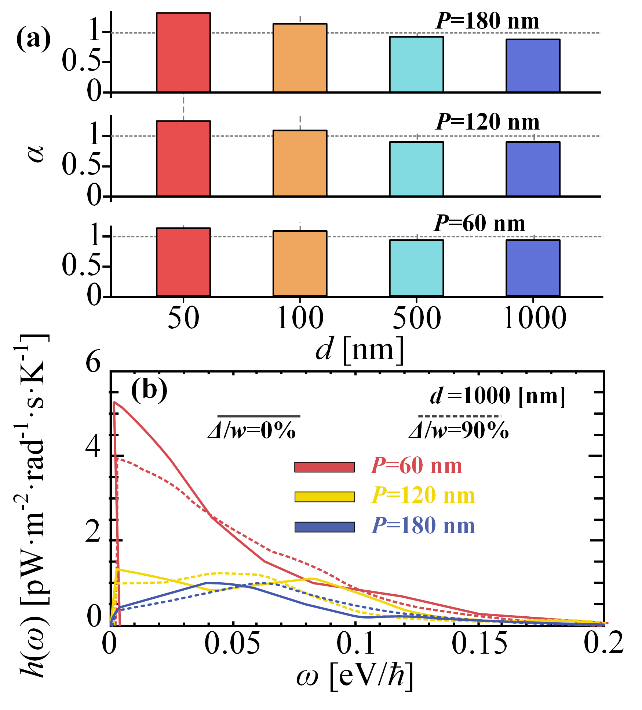


**Fig S5** (a) Enhancement rates *α* = *H*(Δ =0.9 *w*)/*H*(0) of heat transfer coefficients with varying periods at different vacuum gaps. (b) The spectral heat transfer coefficient *h*(*ω*) as a function of the frequency. The different colors correspond to different periods. The solid line shows the conventional mode (Δ =0), and the dotted lines show the interactive thermo-metastructures (Δ/*w* = 0.9).

Nevertheless, we should remark that this interactive thermo-metastructures is not a panacea. Fig. S5 illustrates the HTC enhancement rates *α* and spectral HTC with varying periods at different vacuum gaps. Here, the meta-atomic displacement is fixed at 0.9 Δ/*w*. Even when the period is increased to 180 nm, a notable enhancement in radiative heat transfer can still be achieved between the metastructures within the 50 nm vacuum gap [see Fig. S5(b)]. The results demonstrate that the interactive thermo-metastructures scheme possesses good robustness, which can achieve the manipulation of thermal radiation even in the presence of significant machining accuracy errors. Nevertheless, interactive schemes are not robust to vacuum gaps. When the gap is larger than 500 nm, the interactive scheme restrains the radiative heat transfer performance of the system. The underlying principle of nonlocal metastructures enhanced radiative heat transfer is the extension of thermophoton tunnelling to a larger range of wave vectors, which results in a stronger radiative heat transfer capability. However, electromagnetic waves with large wavevectors are easily filtered out by the vacuum gap and cannot transfer energy from the emitter to the receiver. Therefore, as illustrated in Fig. S5(b), the spectral thermal radiation of the metastructures is not as favorable as that of the local mode when the gap is 1000 nm.

**S4. Thermal photons evolution in designing thermophotonic metastructures with different structure parameters**

To illustrate the impact of alterations on the emergence of nonlocal properties, Fig. S6 depicts the evolution of thermophoton tunnelling at varying Δ. As the meta-atomic displacement increases from 0.2 to 0.5, the thermophoton tunnelling along the *x*-axis is slightly suppressed, yet thermophoton tunnelling towards the *y*-axis extends to a larger range of wavevectors [see Figs. S6(a)-S6(d)]. This phenomenon is the primary cause of the observed increase in the spectral heat flux of the system in Fig. S4(d). Further increasing in the Δ, the effect of the meta-atomic displacement on the wavevector distribution of the thermophoton tunneling is found to be more significant. It demonstrates that a larger Δ (>0.7 *w*) suppresses the wavevector region of stronger thermophoton tunnelling (*ξ*>0.8) as shown in Figs. S6(e)- S6(g). It is noteworthy that a larger meta-atomic displacement promotes the expansion of the bright band of weak thermophoton tunnelling (*ξ*<0.5) into a wider wavevector region, which effectively counteracts the recession of strong thermophoton tunnelling and intensifies the spectral heat flux of the metasurface. However, when the meta-atomic displacement reaches 1 Δ/*w*, it can be observed that, despite the bright thermophoton tunnelling branch extending to a wavevector range close to 60 *k*_0_, the metastructures are no longer able to provide strong thermophoton tunnelling [see Fig. S6(h)]. This, in turn, leads to the non-linear features of the spectral heat flux in Fig. S4(d).


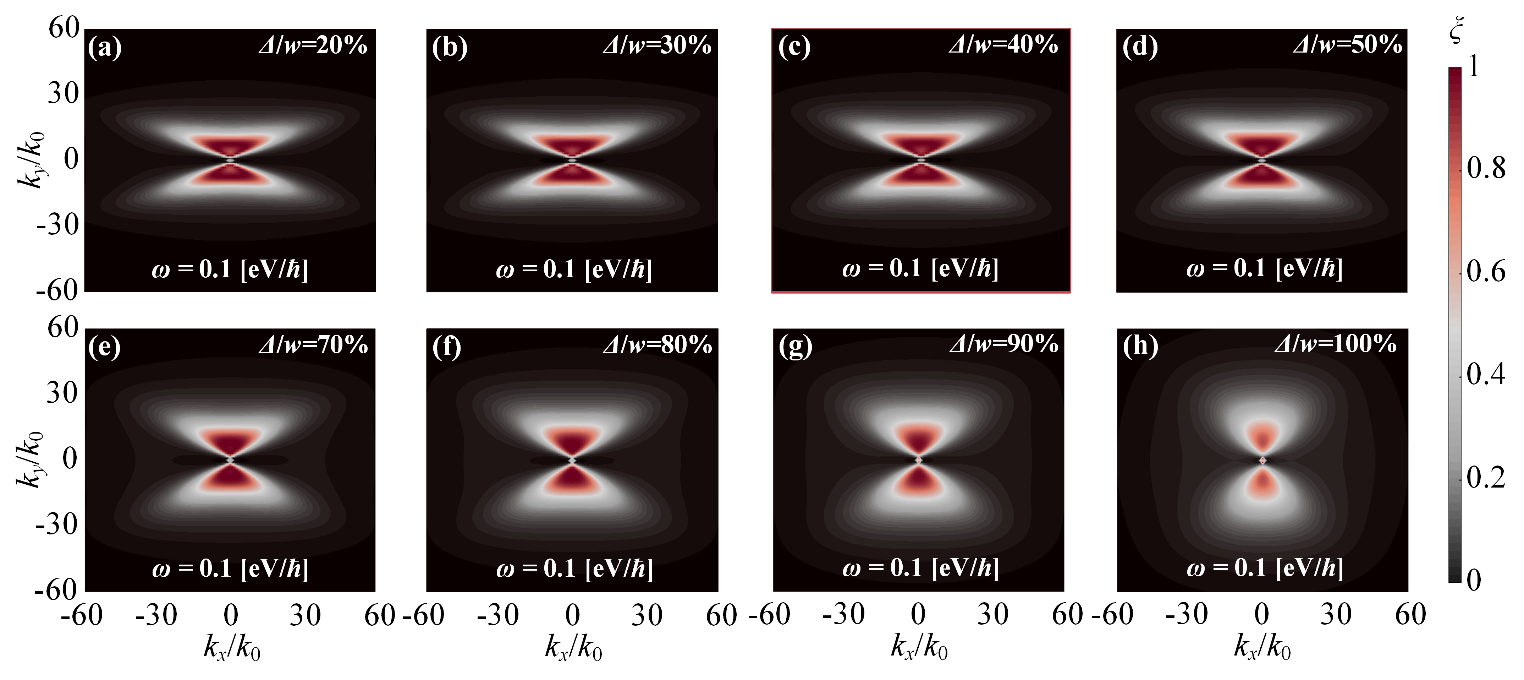


**Fig S6** The evolution of thermophotonic tunnelling probability during increasing meta-atomic displacement, for (a) Δ/*w* = 0.2, (b) Δ/*w* = 0.3, (c) Δ/*w* = 0.4, (d) Δ/*w* = 0.5, (e) Δ/*w* = 0.7, (f) Δ/*w* = 0.8, (g) Δ/*w* = 0.9, and (h) Δ/*w* = 1. The frequency is fixed at 0.1 eV/*ћ*.

**S5. The derivation and result of electromagnetic property with different structure parameter**

It is well established that the fundamental cause of the control of radiative heat transfer can be attributed to the electromagnetic field resonant patterns within it. Consequently, the investigation of electromagnetic resonance modes in metastructures enables a more comprehensive analysis of the impact of nonlocal electromagnetic characteristics induced by meta-atomic displacement on thermophoton tunnelling properties. Fig. S7 also depicts the electric field distribution of this metastructures. When the system is free of meta-atomic displacement the electric field distribution exhibits a clear hyperbolic style in real space. The concave wavefronts of dipole excited hyperbolic surface polariton can be visualized in the near-field distribution of the component *E_z_* (real part). With meta-atomic displacement, although the electric field still has hyperbolic propagation properties along the hypersurface, its field distribution is significantly altered [see Figs. S7(b)- S7(d)]. To show more clearly how this propagation behavior changes, we plot the trend of the distribution when the real part of the electric field is 0 with a dashed line. Increasing the meta-atomic displacement leads to an increasing hyperbolic angle of propagation. This explains why the opening angle of the hyperbolic branch of the thermophoton tunneling coefficient in Fig. S6 changes with increasing meta-atomic displacement.


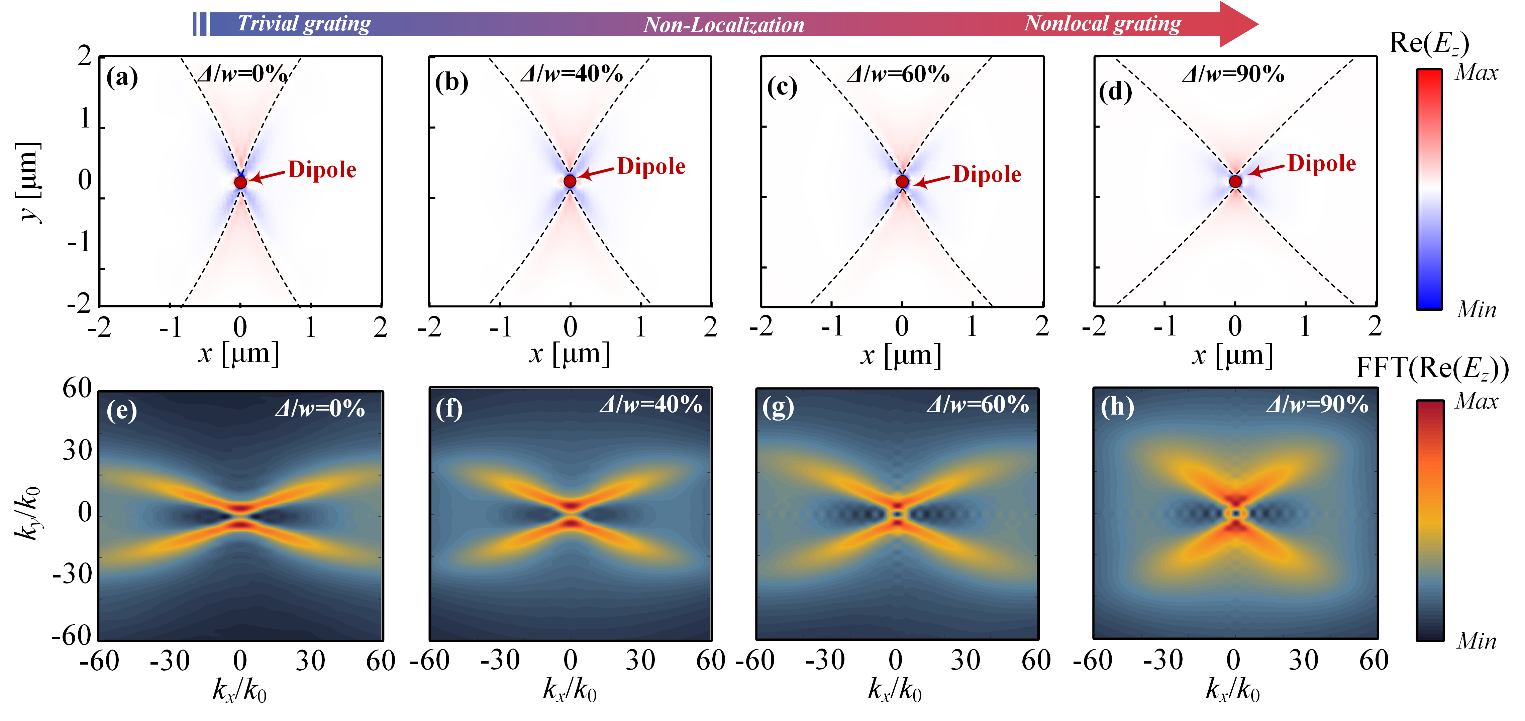


**Fig S7** The real-part of electric field distributions Re(*E_z_*) of metastructures at different meta-atomic displacements in real space: (a) Δ/*w* = 0, (b) Δ/*w* = 0.4, (c) Δ/*w* = 0.6, and (d) Δ/*w* = 0.9. The dotted line represents the distribution of electric field values of zero. The evolution of electric field distributions FFT(*E_z_*) of metastructures in response to different meta-atomic displacements in momentum space: (e) Δ/*w* = 0, (f) Δ/*w* = 0.4, (g) Δ/*w* = 0.6, and (h) Δ/*w* = 0.9. The frequency is fixed at 0.1 eV/*ћ*. The field is excited by a dipole polarized along *z* placed 50 nm above the metastructures.

To better illustrate the effect of the above electromagnetic response on the thermophoton tunneling characteristics, the Fourier transform of the field distribution is shown in Figs. S7(e)-S7(h). For a local metastructures, its dipole-excited field distribution presents an exact hyperbolic bright branch in momentum space. The field energy is concentrated in this hyperbolic branch at this point. However, the emergence of meta-atomic displacement indicates a notable deformation in the field distribution in momentum space. As the meta-atomic displacement increases from 0 to 0.9 Δ/*w*, the bright band branches of the electric field with higher energies (red bright region) are gradually confined to smaller wavevector intervals [see Figs. S7(e)-S7(h)], which is consistent with the evolutionary trend of the thermophoton tunnelling property. Concurrently, the nonlocality induced by meta-atomic displacements results in a more extensive distribution of electric field energy across a broader range of wavevectors in Figs. S7(e)-S7(h). This also explains the considerable enhancement of both the thermophoton tunnelling and spectral thermal radiation of the metastructures when meta-atomic displacements are introduced. Moreover, the augmented displacement also result in a gradual reduction in the opening angle of the hyperbolic bright branch of the electric field, which elucidates the narrow hyperbolic opening angle of the bright branch of the thermophoton tunnelling corresponding to the large meta-atomic displacement in Fig. S5.


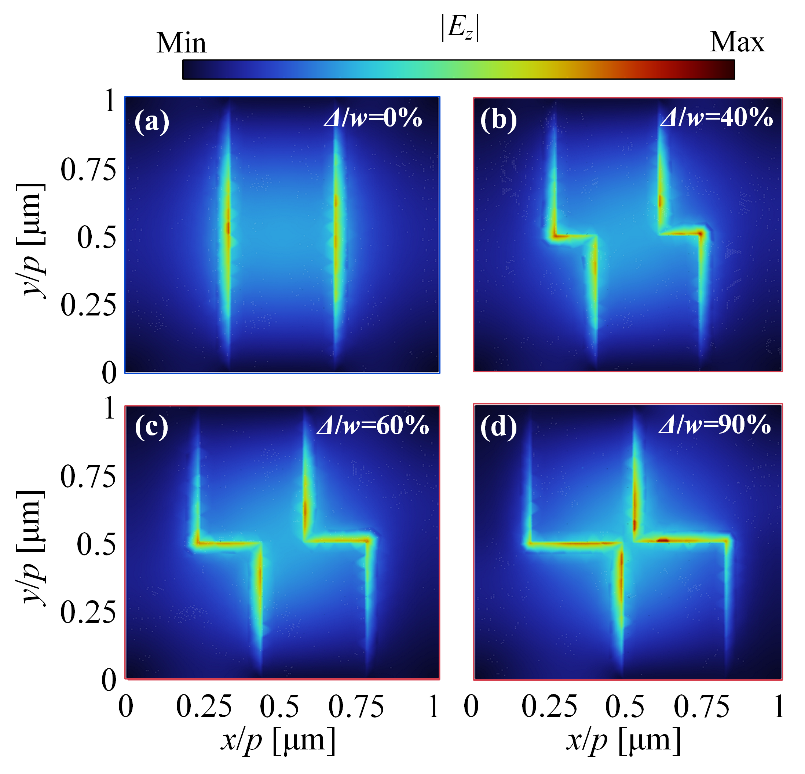


**Fig S8** Electric field profiles (*E_z_*) of cells with meta-atomic displacements of (a) Δ/*w* = 0, (b) Δ/*w* = 0.4, (c) Δ/*w* = 0.6, and (d) Δ/*w* = 0.9, respectively. The field is excited by a dipole polarized along *z* placed 50 nm above the metastructures.

In order to further clarify, next, we calculate the electric field distributions within the patterned cell in Fig. S8. The electric field remains excited by the dipole, which is in the center of the cell at a distance of 50 nm above the surface. It can be observed that the introduction of a meta-atomic displacement results in a notable alteration of the electric field distribution within the cell. Particularly, at the corners generated by meta-atomic displacements, there is a pronounced field concentration, which significantly enhances the surface state strength of the system. This phenomenon is the underlying cause of the capacity of meta-atomic displacement to facilitate the thermophoton tunnelling, as evidenced in the aforementioned results. It demonstrates that when the meta-atomic displacement reaches 0.9 Δ/*w*, the electric field strength in the cell is less than that for a small meta-atomic displacement [see Fig. S8]. This is the fundamental reason why the thermophoton tunnelling intensity of the system shows a significant decrease in the presence of larger meta-atomic displacement in Fig. S5.

**REFERENCES**

[S1] JL Wise and DM Basko. Near field versus far field in radiative heat transfer between two-dimensional metals. *Phys. Rev. B*, 103(16):165423, 2021.

[S2] KF Chen, B Zhao, and SH Fan. Mesh a free electromagnetic solver for far-field and near-field radiative heat transfer for layered periodic structures. *Comput. Phys. Commun.*, 231:163–172, 2018.

[S3] J Dai, SA Dyakov, SI Bozhevolnyi, and M Yan. Near-field radiative heat transfer between metasurfaces a full-wave study based on two-dimensional grooved metal plates. *Phys. Rev. B*, 94(12):125431, 2016.

[S4] S Basu, BJ Lee, and ZM Zhang. Near-field radiation calculated with an improved dielectric function model for doped silicon. *J. Heat Trans-T ASME*, 132(2):023302, 2010.

[S5] XL Liu, B Zhao, and ZM Zhang. Enhanced near-field thermal radiation and reduced casimir stiction between doped-si gratings. *Phys. Rev. A*, 91(6):062510, 2015.

[S6] XH Wu, CJ Fu, and ZM Zhang. Influence of hbn orientation on the near-field radiative heat transfer between graphene/hbn heterostructures. *J. Photon. Energy*, 9(3):032702, 2019.
